# Reduction of Emergency Calls and Hospitalizations for Cardiac Causes: Effects of Covid-19 Pandemic and Lockdown in Tuscany Region

**DOI:** 10.3389/fcvm.2021.625569

**Published:** 2021-03-12

**Authors:** Flavio D'Ascenzi, Matteo Cameli, Silvia Forni, Fabrizio Gemmi, Claudia Szasz, Valeria Di Fabrizio, Maria Teresa Mechi, Matteo Nocci, Sergio Mondillo, Serafina Valente

**Affiliations:** ^1^Division of Cardiology, Department of Medical Biotechnologies, University of Siena, Siena, Italy; ^2^Regional Health Agency of Tuscany, Florence, Italy; ^3^Quality of Care and Clinical Networks, Regional Health Department of Tuscany, Florence, Italy

**Keywords:** lockdown, coronavirus, cardiovascular disorders, acute coronary syndrome, atrial fibrillation, heart failure

## Abstract

**Introduction:** Containment measures were established to flatten the curve of COVID-19 contagion in order to avoid a crash of the healthcare system. However, these measures influenced the rate of hospitalization of cardiac patients. In this study, we aimed to analyse the impact of COVID-19 and the effects of lockdown measures on hospital admissions and alerts of emergency medical system (EMS) for cardiac causes in the Tuscany region.

**Methods:** An observational, retrospective analysis from Italian Tuscany region was conducted. We evaluated consecutive patients contacting EMS or admitted to the 39 Emergency Departments (EDs) in Tuscany for cardiac causes in the first trimester of 2020. Data were compared with the same period in 2018/19.

**Results:** The alerts of EMS for cardiac causes significantly decrease in 2020 and the highest difference between 2018/19 and 2020 was found immediately after national lockdown (Δ = −47.4%, *p* < 0.001). The number of admissions for chest pain in the EDs also decreased, with a maximum difference of −67.6% (*p* < 0.001) vs. 2018/19. The number of hospital accesses for acute coronary syndromes, atrial fibrillation, and heart failure in the EDs significantly decreased in 2020 as compared to 2018/19 (maximum Δ = −58.9%, *p* < 0.001; maximum Δ = −63.0%, *p* < 0.001; maximum Δ = −72.7%, *p* < 0.001, respectively).

**Conclusions:** A significant decrease in the contacts to EMS for cardiac causes and in cardiac diagnoses was observed during the first trimester of 2020. Fear of contagion has likely played a relevant role. The lesson learnt from first wave of COVID-19 pandemic suggests that appropriate public information strategies and re-education of people are essential.

## Introduction

The pandemic caused by COVID-19 has been associated with thousands of deaths worldwide and multiple cardiovascular risk factors and cardiac disorders have been recognized as high-risk conditions (1). The rapidly increasing number of patients affected by COVID-19 requiring hospitalization has imposed a relevant problem of sustainability for the healthcare system. Accordingly, during the first wave of COVID-19 pandemic, the Italian government has imposed measures promoting social distancing and a stepwise strategy starting from the quarantine for some Italy regions with subsequent lockdown measures adopted for the entire nation as of 11 March (https://www.gazzettaufficiale.it/eli/id/2020/03/08/20A01522/sg, https://www.gazzettaufficiale.it/eli/id/2020/03/11/20A01605/sg). Although these strategies were aimed to flatten the curve of the contagion in order to avoid a crash of the health care system, these measures have significantly influenced the rate of hospitalization of cardiac patients and changes in the pattern of hospital admissions have been noted, particularly in the Northern regions of Italy (2–4).

In this study, we aimed to analyse the epidemiologic impact of COVID-19 and the effects of lockdown measures on the contacts to emergency medical system (EMS) and hospital visits to the emergency department for cardiac causes for the entire Tuscany region. The number of final diagnoses of acute coronary syndrome (ACS), heart failure (HF), and atrial fibrillation (AF) was also considered. These data were compared with the trends observed in the same time frame of the previous 2 years.

## Methods

We conducted an observational, retrospective analysis from the Tuscany region aimed at evaluating the number of patients contacting the EMS for cardiac problems and symptoms, not occurring during COVID-19 infection (i.e., angina, arrhythmias, syncope, chest pain, etc.), with high dispatch priority, established by nurse triage, and the number of consecutive patients admitted to the Emergency Departments for cardiac causes, analyzing the final number of diagnoses of ACS, HF, and AF. In Tuscany there were 3.73 million inhabitants and 39 Emergency Departments that performed 1,537,031 visits (data for the year 2019). The period of observation lasted 3 months, i.e., the first trimester of 2020, from the 1st of January 2020 to the 31th of March 2020. This period was selected taking into account that the first cluster of cases of COVID-19 was identified in Italy the 20th of February and that lockdown measures were adopted for the entire nation as of 11th March. Weekly data observed during this period were compared to the trends observed in the same time frame of 2018 and 2019. Although the first cluster of cases of COVID-19 was identified in Italy the 20th of February 2020, the entire first trimester of 2020 was included in this analysis to show also pre-COVID 19 data and to demonstrate that differences in the rate of hospitalization in March were not due to physiologic fluctuations due to epidemiologic factors. A sub-analysis was also performed dividing the first trimester 2020 into three different periods, according to the events occurred during this trimester: 1st January-20th February; 21th February-10th March; 11th March-31th March. Number of accesses to Emergency Departments for stroke and sepsis were also analyzed.

The regional information systems of pre-hospital and hospital EMSs and hospital admission abstracts were used as data sources. These databases include calls to EMS, visits to emergency departments and hospital admissions in Tuscany region. In these data each individual has a unique and anonymous identifier that enables complete record linkage at individual level.

Although the comparison of the rate of mortality between the first trimester 2020 and 2018/2019 was beyond the primary scope of this study, the in-hospital mortality for patients admitted for ACS and HF was also analyzed. The rate of hospitalizations for patients admitted to the Emergency Departments and the number of patients with ACS and HF admitted to the intensive care units of the Tuscany Region during the hospitalization was also analyzed for the entire period. Data were analyzed and were checked for missing or contradictory entries and for values out of normal range by Regional Health Agency of Tuscany.

This study was conducted in accordance with the Helsinki Declaration. According to the Italian legislation (legislative decree 211/2003) and the regional procedures, the study does not need ethic approval as it is a purely observational study on routine collected anonymous data. Furthermore, because this was an observational retrospective study, patients had already been treated when the study protocol was written; therefore, it could not have modified their life-trajectories or care pathways in any way.

### Statistical Analysis

Mean values of data obtained in the first trimester of 2018 and 2019 were calculated and compared with data collected in the same period of 2020. Ninety-five percentage confidence intervals of values observed in 2018-19 were calculated using Poisson model for each week and for the three periods considered in the study. Differences between periods of observation for 2018/2019 and 2020 were expressed as Δ and statistical significance was tested using Poisson models. The statistical significance was set for a two-tailed *p*-value < 0.05. Data were collected using Excel software (version 16.35 2019, Microsoft Corporation, Redmond, USA). The statistical software Stata 14 SE (StataCorp LP, College Station, Texas) was used for the data analyses.

## Results

**Figure 1:**
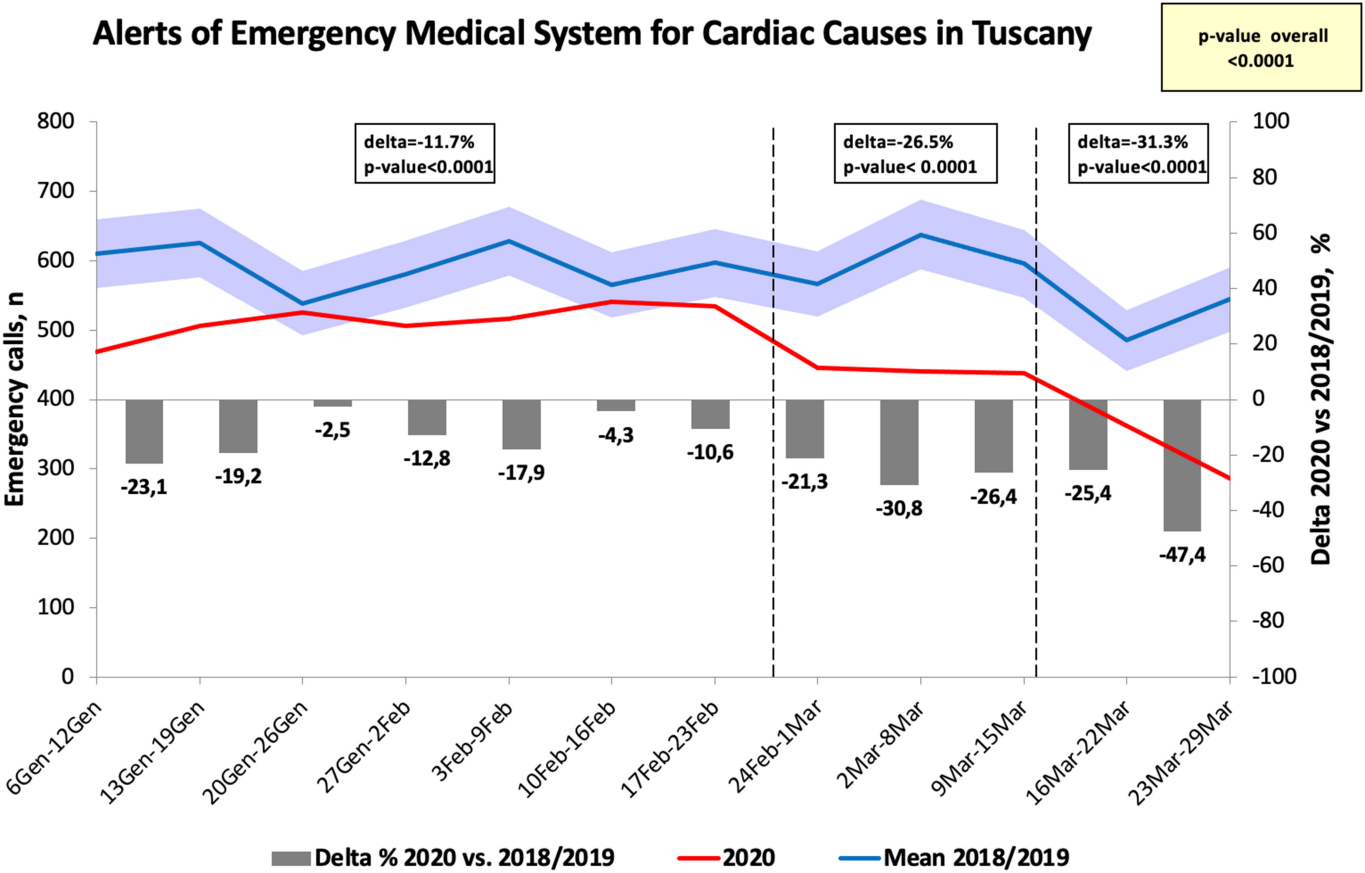
Number of calls to the emergency medical system for cardiac causes in Tuscany. Data obtained in the first trimester were compared to data observed in 2018 and 2019 (an average value of this data was observed). For all the figures data were reported for each week separately (from the 6th of January to the 30th of March) and the delta between 2020 and 2018/2019 was reported and expressed by gray columns as percentage as well as delta and *p*-values for the three different periods.

A significant decrease in contacts of EMS by the patients for cardiac causes was found between 2019 and 2020, see Figure 1. The highest difference was found 1 week after the national lockdown was imposed (Δ = −47.4% as compared to the same week of the previous years, *p* < 0.001).

**Figure 2:**
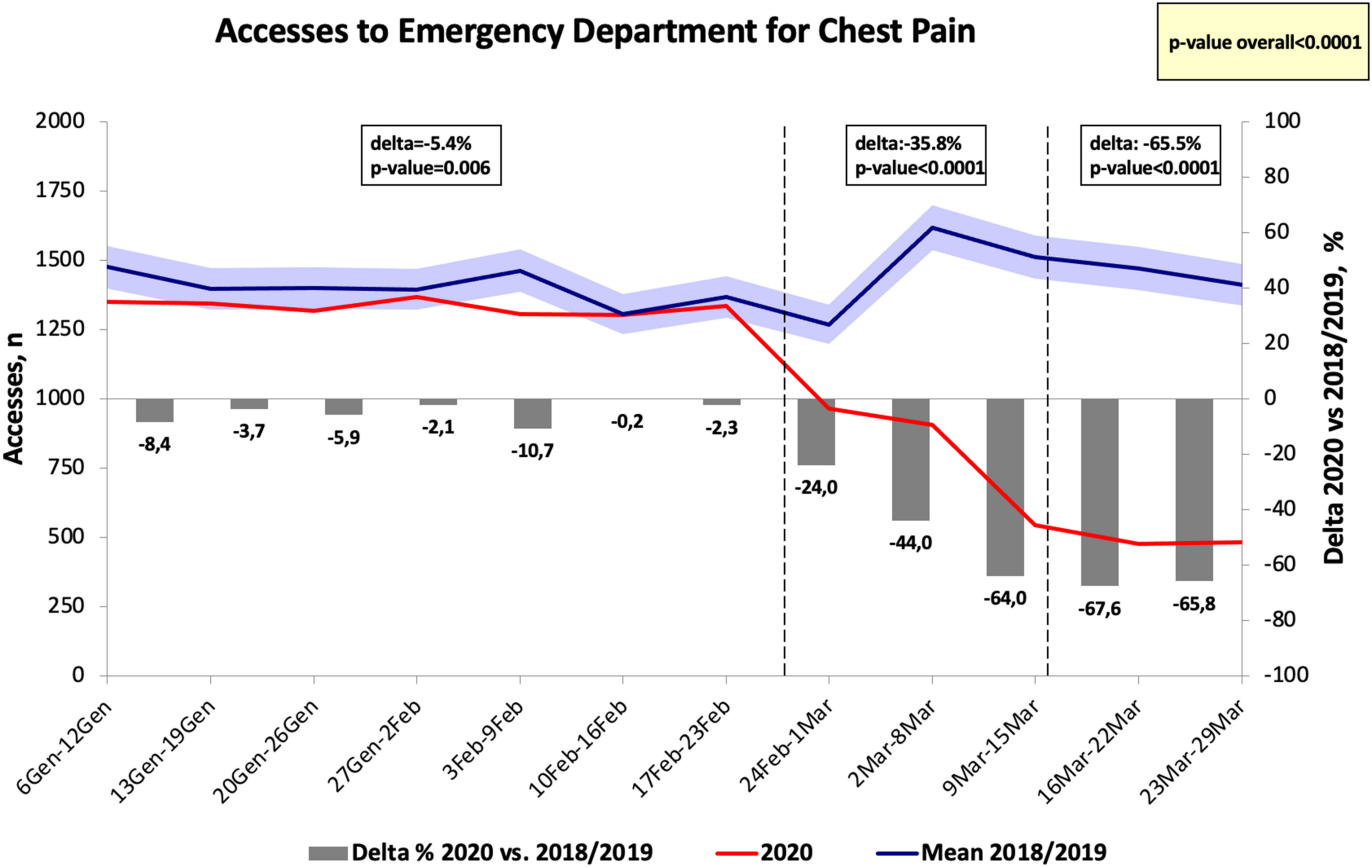
Number of accesses to emergency department for chest pain in Tuscany.

**Figure 3:**
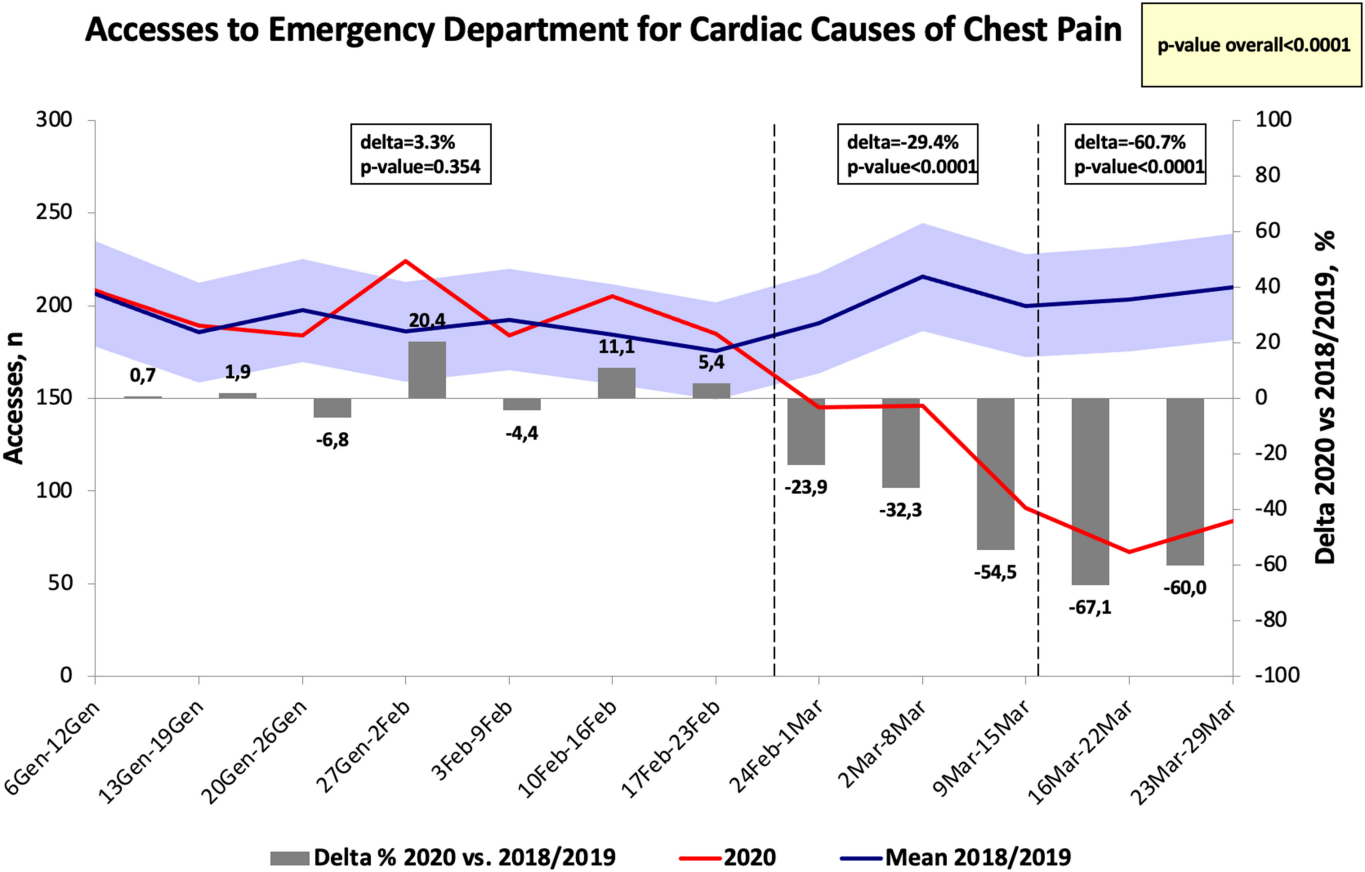
Number of accesses to emergency department for cardiac causes of chest pain in Tuscany.

The numbers of hospital visits for chest pain in the Emergency departments in Tuscany significantly decreased in 2020 as compared to 2018 and to 2019, reaching a Δ at the end of the week between 24 February-01 March of −24.0% (*p* < 0.01 vs. the same period of the previous years), see Figure 2. The week after the national lockdown, the number of visits for chest pain significantly dropped to −67.6% as compared to the same time frame of 2018 and 2019 (*p* < 0.001) and it represented the highest difference found between 2020 and the previous years. While no significant differences were found before the 24th of February for the visits to the Emergency departments for cardiac causes of chest pain (*p* = 0.354), they significantly decrease after this first period (see Figure 3).

**Figure 4:**
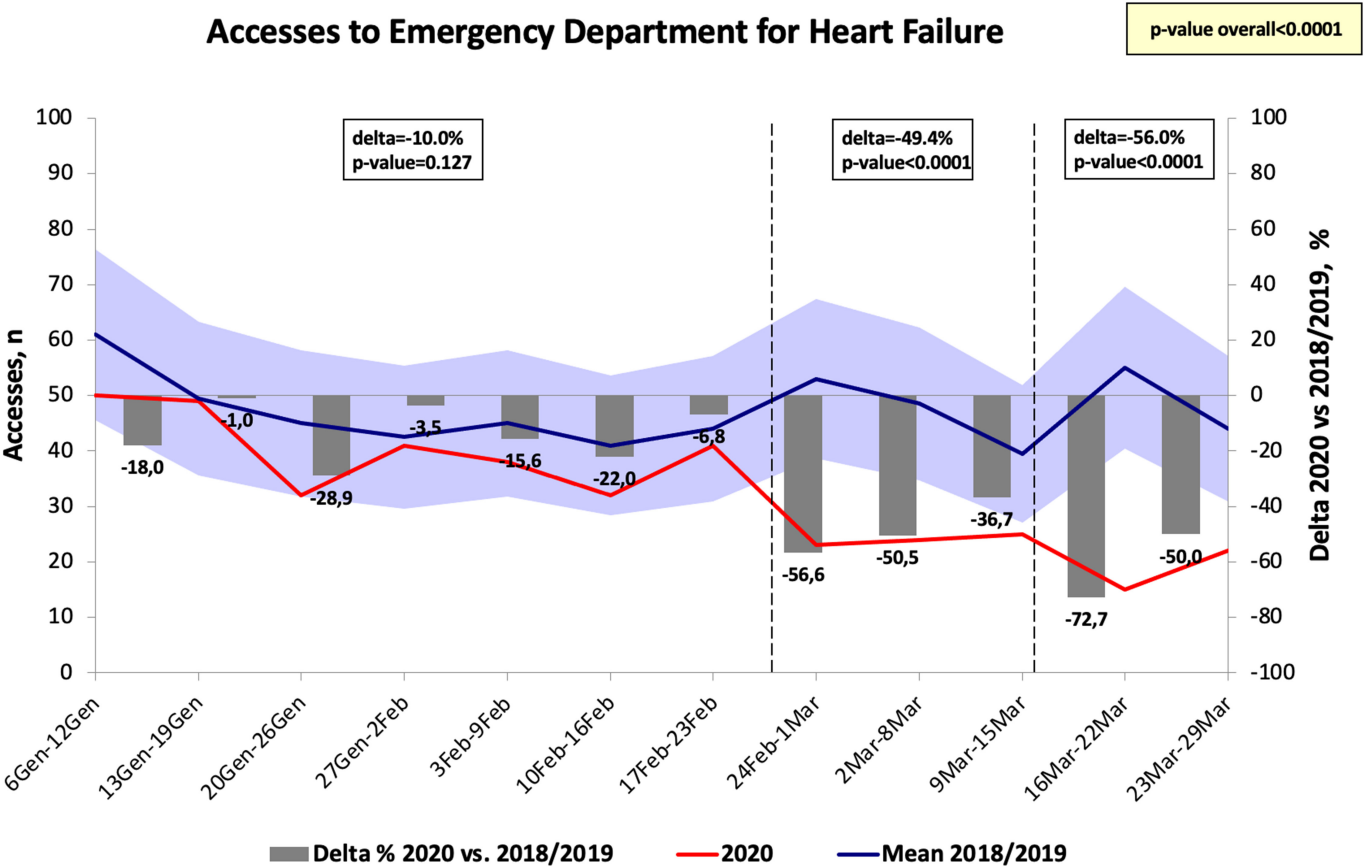
Number of accesses to emergency department for acute coronary syndrome in Tuscany.

**Figure 5:**
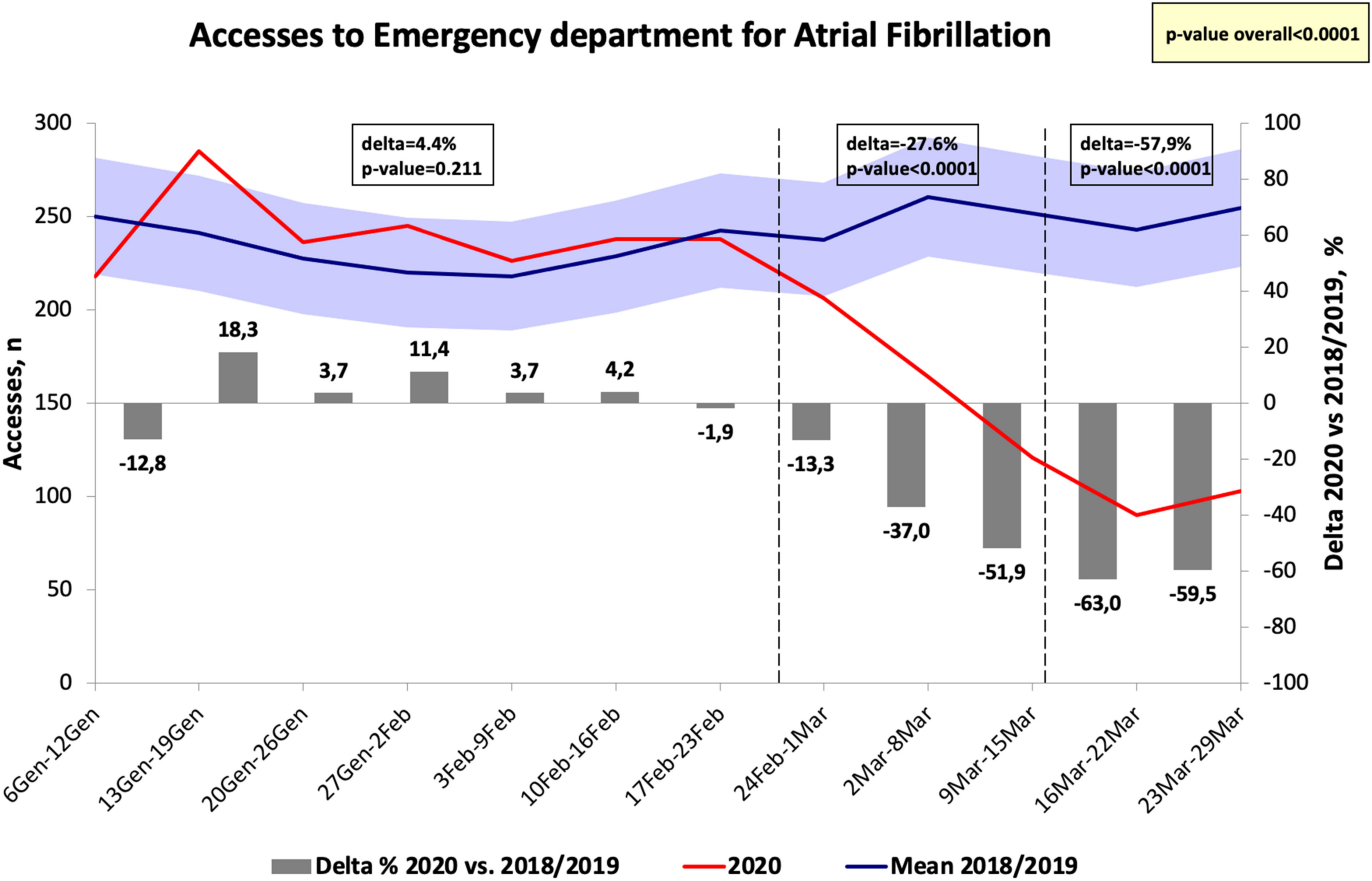
Number of accesses to emergency department for atrial fibrillation in Tuscany.

**Figure 6:**
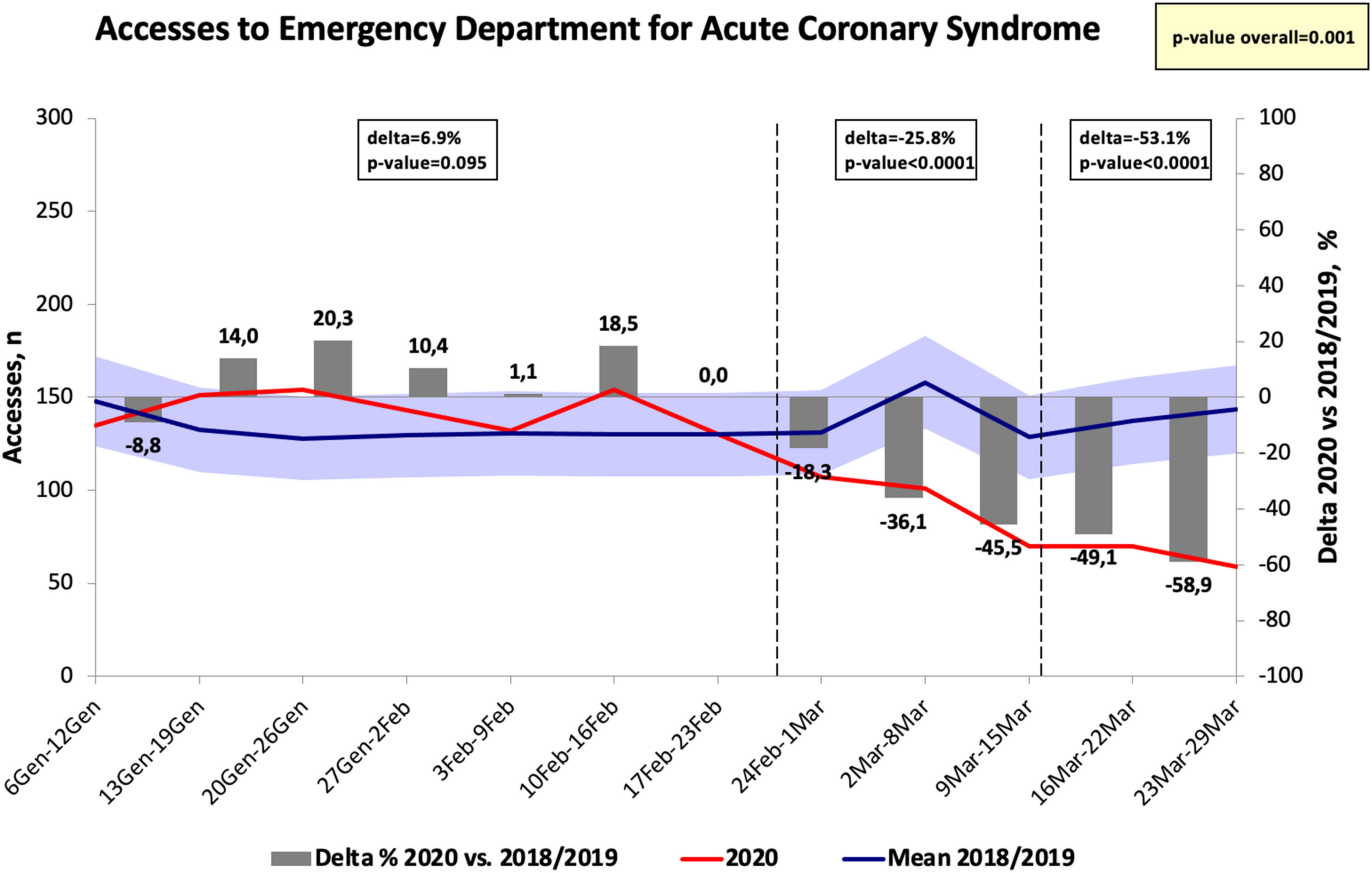
Number of accesses to emergency department for heart failure in Tuscany.

The number of hospital visits for ACS in the Emergency departments significantly decreased at the end of February as compared to 2018 and 2019 (Δ = −18.3%, *p* < 0.05) and the greatest difference was identified at the end of March 2020 (Δ = −58.9%, *p* < 0.001) (Figure 4). Similarly, the diagnosis of AF in the Emergency departments significantly decreased at the end of February 2020 as compared to the same period in 2018 and 2019 (*p* < 0.05), reaching the greatest difference in the week after the national lockdown (Δ = −63%, *p* < 0.001) (Figure 5). The diagnosis of HF significantly decreased during COVID-19 pandemic, reaching the greatest difference in comparison with 2018/2019 data 1 week after the declaration of national lockdown (Δ = −72.7%, *p* < 0.001, Figure 6). The number of accesses to Emergency Departments due to stroke or sepsis were also decreased during the first wave of COVID-19 pandemic as compared to 2018 and 2019 (see Supplementary Figures 1, 2).

The rate of hospitalizations for patients admitted to the Emergency Departments did not differ between 2020 and 2018/2019 (overall *p*-value = 0.68) for ACS and for HF (overall *p*-value = 0.49). The in-hospital mortality for patients suffering from an ACS did not differ between the first trimester 2020 and the first trimester of 2018 and 2019 (overall *p*-value = 0.166). During the three different periods no significant differences were observed (*p* = 0.71, *p* = 0.0.92, and *p* = 0.364, respectively). Among the patients admitted for ACS to the hospitals of the Tuscany Region, the number of patients requiring hospitalization in an intensive care unit did not differ between the first trimester 2020 and 2018/2019 (overall *p*-value = 0.11).

The in-hospital mortality for HF did not differ between 2020 and 2018/2019 (overall *p*-value = 0.102), with no differences among the three different periods (*p* = 0.053, *p* = 0.269, and *p* = 0.208, respectively). Among the patients admitted for HF to the hospitals of the Tuscany Region, the number of patients requiring hospitalization in an intensive care unit did not differ between the first trimester 2020 and 2018/2019 (overall *p*-value = 0.29).

## Discussion

The main finding of the present study is that a marked decrease in the number of patients alerting the EMS and visiting the Emergency departments for cardiac causes were observed in Tuscany after the diagnosis of the first cluster of COVID-19 cases in Italy and particularly after the national lockdown, as compared to the same time frame of the previous years (i.e., 2018 and 2019). As a consequence, the number of ACS and AF diagnosed significantly decreased as compared to the same period of the previous years. Notably, the trend demonstrates a significant drop after the 20th of February and after the 11th of March 2020, i.e., after the first case diagnosed and after the national lockdown. Multiple factors may have affected the rate of visits and hospitalization for cardiac causes during the most dramatic periods of COVID-19 pandemic, as demonstrated also by the unpredictable reduction in hospitalizations for other causes, such stroke and sepsis. However, these findings indirectly suggest that the fear of contagion at the hospital probably have discouraged the patients to alert the EMS during the first wave of COVID-9 pandemic, particularly after the media diffused the news that infection was spread across hospitalized patients and healthcare personnel. The concerns raised by the mass media on the high mortality rate of COVID-19 pneumonia further discouraged patients with cardiac conditions to contact the EMS. As reported by De Rosa et al. (2), a second hypothesis can be that the emergency medical system was focused on COVID-19. However, our study demonstrates that the number of calls to the EMS significantly decrease during this dramatic period; while variations in the rate of ACS and cardiac disease have been demonstrated (5, 6) and cannot be definitely excluded, the marked difference between the same periods of 2018/2019 and 2020, reaching even more than −65% reduction in the visits, suggest that patients intentionally decided not to alert the EMS or to go to the hospital, irrespective of their cardiac conditions and their symptoms. Unfortunately, this phenomenon was not confined to Italy, but sharp drops in the numbers of persons seeking emergency medical care was observed also in United States, with the total number of US ED visits being 42% lower during the early pandemic period than during the same period a year earlier (7), and in Thailand (−36%) (8). Notably, also in US the decrease in ED visits for acute life-threatening health conditions was observed immediately before and after declaration of the COVID-19 pandemic as a national emergency (9). In agreement with our findings, also Wongtanasarasin et al. observed that the national lockdown in Thailand was associated with a significant reduction in average daily ED visits (8).

A reduction in ACS activations was reported also by US cardiac catheterization laboratories and was noticed also in Spain (10, 11) and in a recent survey conducted by the European Society of Cardiology the respondents declared a reduction in the admission of patients with ACS >40% (12). In Italy, a reduction in the rate of hospital admissions for ACS was reported by De Rosa et al. for the week 12–19 March (2), by Toniolo et al. (3), and by De Filippo et al. (4). Notably, the study by De Rosa was a national registry with analysis confined to 1 week while the other two articles included centers in the Northern part of Italy, i.e., the most affected by COVID-19 pandemic. Indeed, Lombardy and Piedmont regions had 89,526 and 30,758 confirmed cases of COVID-19, respectively, while in Tuscany 10,122 cases were diagnosed (http://opendatadpc.maps.arcgis.com/apps/opsdashboard/index.html#/b0c68bce2cce478eaac82fe38d4138b1 last access, 06/05/2020). In this study we extended the time frame of observation reporting the data of the first trimester 2020 from a different region of Italy, i.e., the Tuscany, and we demonstrated that a low rate of contacts to EMS during this pandemic was observed also in regions of Italy less affected by the pandemic. We found that the reduction in admission was observed also for patients with heart failure, with a delta of −56% for the last period of observation in comparison with 2018 and 2019 (*p* < 0.0001), in agreement with data reported by Severino et al. and demonstrating a reduction of admission during the lockdown (13). These findings suggest that the ubiquitous presence of COVID-19 news on the mass media and social media and the lack of verified information have contributed to the perception of unsafe hospitals, even if hospital were not overwhelmed by the COVID-19 emergency, as in Tuscany, and an underestimation of mortality and morbidity risks due to cardiac conditions. Indeed, as demonstrated by Barbieri et al., the reduction in hospital admissions observed in 2020 ad compared to the same period of 2019 was associated with increased mortality (14).

Finally, we found in this study that, for the first trimester 2020, the in-hospital mortality did not differ for patients admitted for ACS and for HF, in comparison with the first trimester of 2018 and 2019. Furthermore, the number of patients with ACS and HF requiring hospitalization in an intensive care unit did not differ. Although the impact of the decrease in the number of hospitalizations and visits to the Emergency Departments on the cardiovascular mortality was not the primary scope of this study, these findings suggest that patients were treated with similar standards before and during the first wave of COVID-19 pandemic and with similar outcomes. However, the low rate of hospitalizations for ACS and AF may represent a warning alert for the future development of cardiac and cerebrovascular complications, such as end-stage heart failure, sudden death, or transient ischemic attack and stroke and the negative effects of this marked impact on the pattern of hospitalizations will likely be seen in the next future. Further studies extending the period of observation are needed to report a comprehensive analysis of this phenomenon. Furthermore, the negative impact of the reduction in hospitalization for cardiac causes may have cause an increase in out-of-hospital mortality. Unfortunately, these data were not available.

The present data further strengthen the need of adequate public information policies to reinforce the importance of timely care for medical emergencies. Furthermore, the lesson learnt from the first wave of COVID-19 pandemic suggests that the community of healthcare professionals should continue re-educating the general population to recognize early cardiac symptoms (2) and to be confident with the national healthcare system in case of hospitalization.

### Limitations

In this study we observed a dramatic decrease of hospital admissions and emergency contacts, primarily due to the fear of contagion. Although the fear of contagion likely was the primary mechanisms leading to the reduction of hospital admissions, a multiplicity of factors, rather than a unique mechanism, contributed to this phenomenon. As reported by De Rosa et al. (2), we cannot completely exclude that a true reduction in the incidence of acute cardiovascular disease as the potential result of low physical stress and widespread prevalence of the resting state during the quarantine, especially in the initial phase of the social containment, might have partly contributed to the lower number of admissions.

Although patients affected by SARS-CoV-2 were excluded from the final analysis, we cannot definitively exclude that some cardiac symptoms suffered from patients contacting the EMS may be related to cardiac consequences of COVID-19 infection.

## Conclusions

In Tuscany a significant decrease in the contacts to EMS for symptoms and disease related to cardiac causes and in the hospitalization rate for ACS, AF, and HF was observed during the COVID-19 pandemic. In the comparison with the same period of the previous years, the greatest difference was identified after the first case of COVID-19 in Italy and after the national lockdown. Fear of contagion among the patients has likely played the most relevant role. Therefore, the lesson learnt from the first wave of COVID-19 pandemic suggests that appropriate public information strategies are essential for a proper management of cardiac patients and a re-education of general population to recognize cardiac symptoms and life-threatening cardiovascular disorders and the consequent need of hospitalization should be guaranteed.

## Data Availability Statement

The raw data supporting the conclusions of this article will be made available by the authors, without undue reservation.

## Author Contributions

FD'A, SM, and SV contributed to the conception of the study while FD'A, SF, FG, and MN contributed to the design of the study. FD'A wrote the manuscript. MC, SF, FG, CS, VD, MM, SM, and SV critically revised the manuscript. All the authors gave the final approval and agrees to be accountable for all aspects of work ensuring integrity and accuracy.

## Conflict of Interest

The authors declare that the research was conducted in the absence of any commercial or financial relationships that could be construed as a potential conflict of interest.

## Supplementary Material

Supplementary Figure 1 Number of accesses to emergency department for stroke in Tuscany.

Supplementary Figure 2 Number of accesses to emergency department for sepsis in Tuscany.
