# Supplementary figures and images for: Reduction of Emergency Calls and Hospitalizations for Cardiac Causes: Effects of Covid-19 Pandemic and Lockdown in Tuscany Region
Source: Front Cardiovasc Med. 2021 Mar 12;8:625569. doi: 10.3389/fcvm.2021.625569 (PMC7994258; doi:10.3389/fcvm.2021.625569)

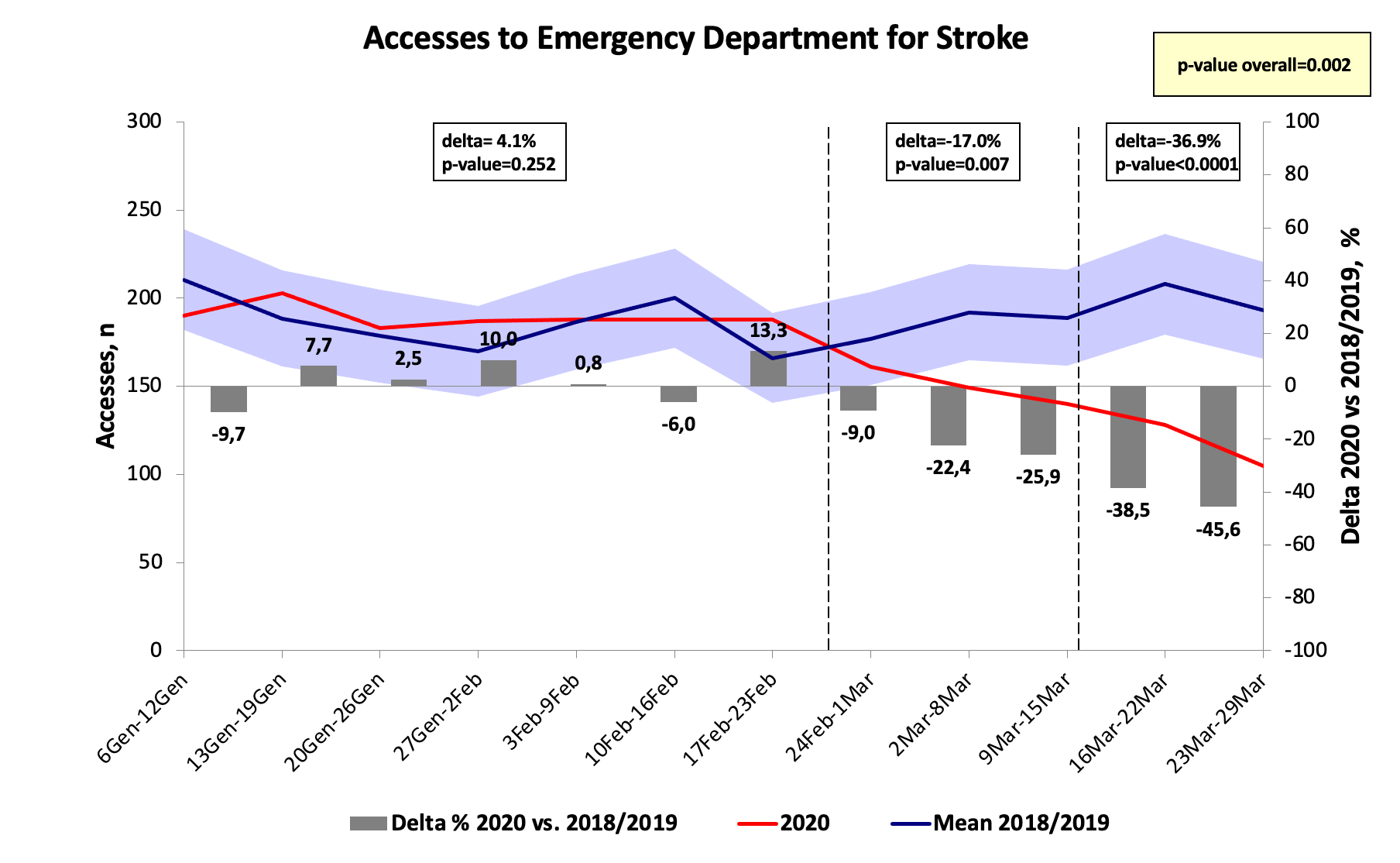

Supplement: Supplementary Figure 1 — Number of accesses to emergency department for stroke in Tuscany. [file Image_1.TIF]

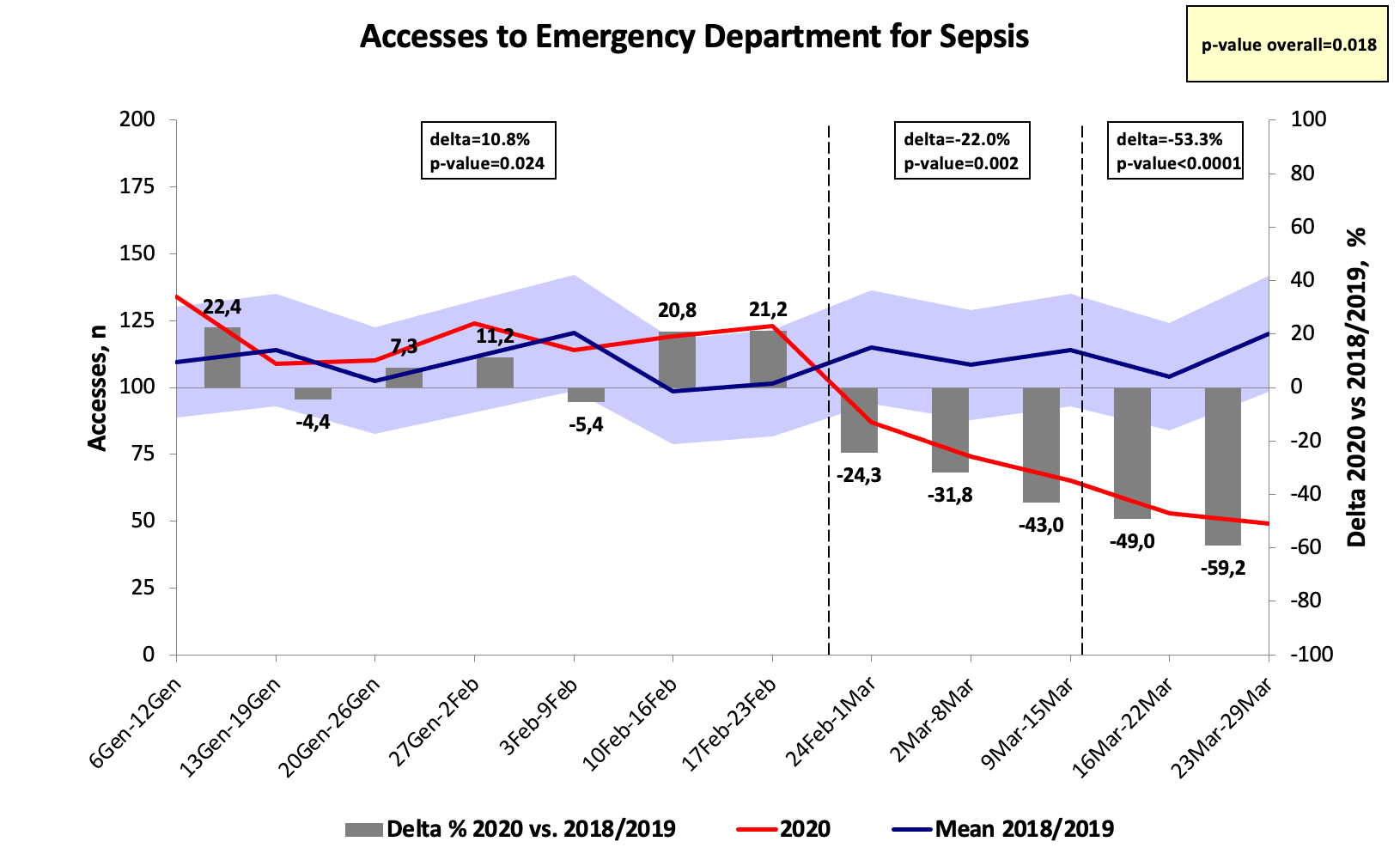

Supplement: Supplementary Figure 2 — Number of accesses to emergency department for sepsis in Tuscany. [file Image_2.TIF]
